# The effect of dietary phosphorus load and food matrix on postprandial serum phosphate in hemodialysis patients: a pilot study

**DOI:** 10.12688/hrbopenres.13382.1

**Published:** 2021-11-10

**Authors:** Fiona Byrne, Barbara Gillman, Brendan Palmer, Mairead Kiely, Joseph Eustace, Patricia Kearney, Fred Davidson, Frances Shiely

**Affiliations:** 1 Department of Nutrition & Dietetics, Cork, Cork University Hospital, Cork, T12 DC4A, Ireland; 2 Department of Renal Medicine, Cork University Hospital, Cork, T12 DC4A, Ireland; 3 Health Research Board, Clinical Research Facility Cork, University College Cork, Cork, T12 WE28, Ireland; 4 Department of Nutrition & Dietetics, Mater Misericordiae University Hospital, Dublin, D07 R2WY, Ireland; 5 School of Public Health, University College Cork, Cork, T12 XF62, Ireland; 6 School of Food and Nutritional Sciences, University College Cork, Cork, T12 T656, Ireland; 7 Cork Public Analyst's Laboratory, St. Finbarr's Hospital, Cork, T12 XH60, Ireland

**Keywords:** Hyperphosphataemia, Phosphorus, Potassium, Diet, Haemodialysis

## Abstract

**Background: **Potential dietary strategies for controlling hyperphosphataemia include the use of protein sources with lower phosphorus bioavailability such as pulses and nuts, focus on phosphorus to protein ratios and the avoidance of all phosphate additives.

**Methods:** We conducted a controlled crossover feeding study in 8 haemodialysis (HD) patients to investigate the acute postprandial effect of a modified versus standard low phosphorus diet for one day on serum phosphate, potassium and intact parathyroid levels in prevalent HD patients. Each participant consumed the modified diet on one day and the standard diet on a second day one week apart. The modified diet included beef and less dairy, with a lower phosphorus to protein ratio, as well as plant-based protein, whole grains, pulses and nuts containing phytates which reduces phosphorus bioavailability. Both diets were tailored for each participant to provide 1.1g protein/kg ideal body weight. Participants provided fasting bloods before breakfast, a pre-prandial sample before the lunch time main meal and samples at one-hour intervals for the four hours after the lunch time main meal, for analysis of phosphate, potassium and intact parathyroid hormone (iPTH).

**Results: **At four hours post the lunch time main meal on each study day, individuals on the modified diet had serum phosphate readings 0.30 mmol/l lower than when on the standard diet (p-value = 0.015, 95% confidence interval [CI] -0.57, -0.04). The corresponding change in serum potassium at four hours was a decrease of 0.675 mmol/l (p-value = 0.011, CI -1.25, -0.10).

**Conclusions**: Decreases in both serum phosphate and serum potassium readings on a modified low phosphorus diet encourage further larger studies to explore the possibility of greater food choice and healthier plant-based diets in HD patients.

**ClinicalTrials.gov registration: **NCT04845724 (15/04/2021)

## Introduction

Hyperphosphataemia is associated with increased mortality in end stage kidney disease ^1, 2^. Higher phosphate levels, even within normal range are associated with increased mortality in those in the earlier stages of chronic kidney disease (CKD), and in patients with normal renal function ^3– 8^.

Hyperphosphataemia is a major therapeutic target in the management of chronic kidney disease mineral and bone disorder (CKD MBD) and the use of phosphorus restricted diets is well established in the treatment of hyperphosphataemia in patients with CKD MBD ^9^.

Few high quality trials have examined the impact of the dietary restriction of phosphorus and the evidence base is therefore weak ^10^. Efforts to control total phosphorus intake may lead to the unnecessary restriction of higher fibre foods such as pulses and whole grains where the phosphorus is largely bound to phytate and thus unavailable for absorption ^11^. Serum phosphate is not an ideal biomarker to use as an outcome measure in healthy or CKD populations. Circadian rhythm ^12– 16^ and fasting versus non fasting measurements ^14, 15, 17– 19^ can effect serum phosphate, challenging the interpretation of concentrations.

The mechanism of dietary phosphorus absorption remains poorly understood ^20, 21^ and we have limited data on postprandial changes in serum phosphate level ^14, 22, 23^.

Following a review of emerging evidence, a national working group of Irish dietitians identified three strategies to improve the management of dietary phosphorus restriction; (1) the introduction of plant protein in the form of pulses, nuts and whole grains, (2) consideration of protein intake in terms of load as well as the phosphorus to protein ratio of different foods and (3) reduction of phosphate additive-containing foods ^24, 25^. The rationale for this study was to examine the potential benefit of modifying the standard diet as used in clinical practice, to improve the quality and choice of foods allowed. This echoed a growing call internationally to liberalise the renal diet and to include vegetarian options, whilst acknowledging the need for careful monitoring of potential risks such as hyperkalaemia ^26– 30^.

The feeding study described here was conducted to assess the effect of these proposed changes in a controlled environment over a short time period, providing insight into acute biochemical changes that occur in the postprandial phase.

## Methods

### Study design

**Figure 1.**
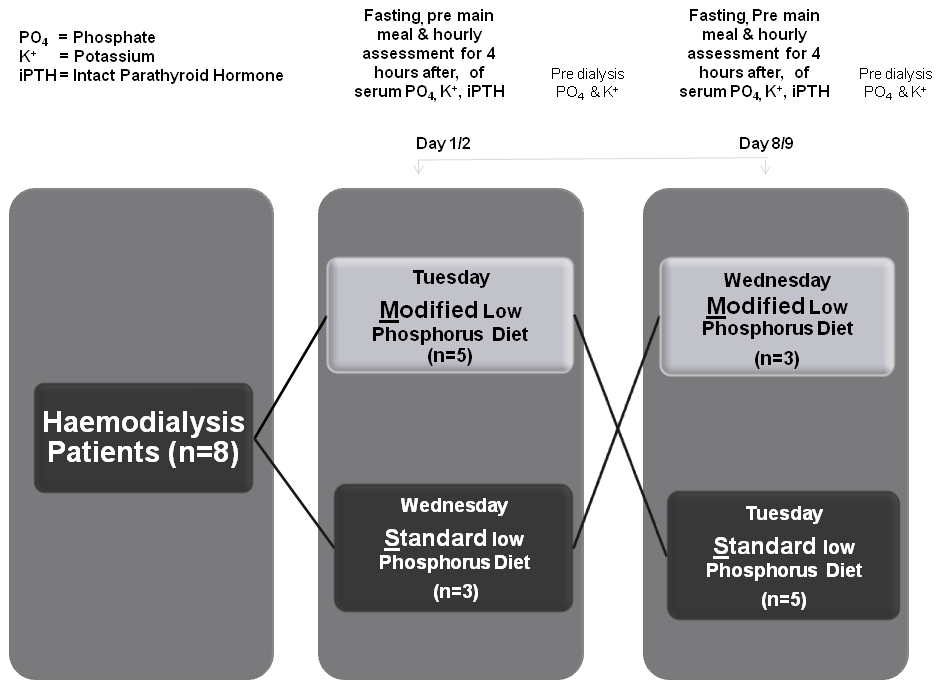
Study design and outcome assessments. In total, five participants attending the first day were assigned to the modified diet, and three participants attending the following day received the standard diet. All participants returned the following week and received the opposite diet. On each day participants had fasting bloods on arrival, before the main midday meal and hourly following completion of the meal for 4 hours. Pre dialysis bloods were taken the following day before dialysis.

We conducted a crossover study comparing the effect of two diets, a standard low phosphorus diet and a modified low phosphorus diet as described in Figure 1. The trial was registered at ClinicalTrials.gov on 15 ^th^ April 2021 (NCT04845724).

### Ethical considerations

All participants provided informed written consent and the protocol was approved by the Clinical Research Ethics Committee (ECM 06/2021 PUB) and conformed with the 1975 Declaration of Helsinki, as revised in 2013.

### Procedure

**Figure 2.**
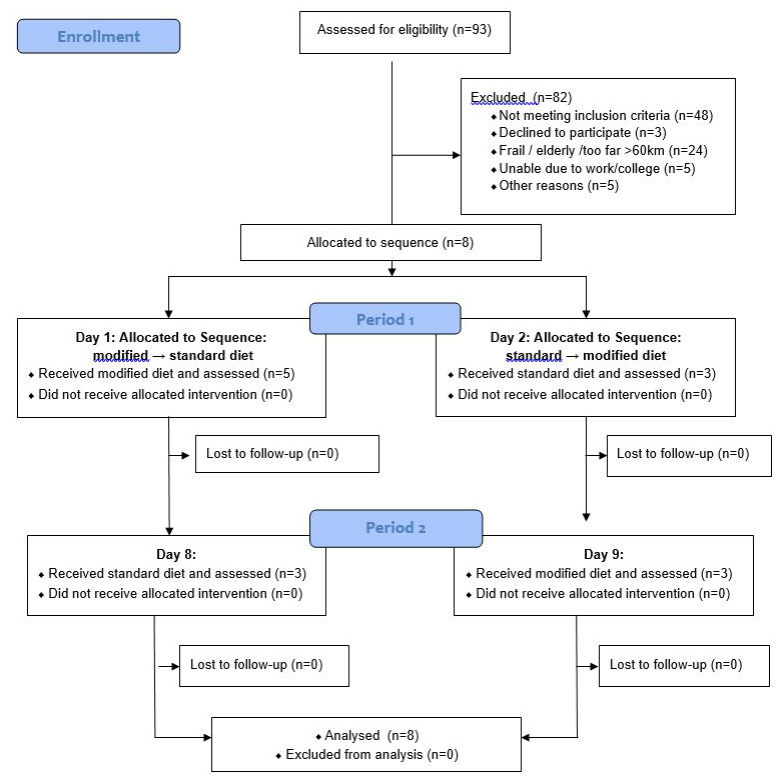
Study flow chart.

Participants were recruited from a university hospital dialysis unit. All patients on dialysis, three times per week, who attended Monday and Wednesday or Tuesday and Thursday, were included screened and assessed, resulting in a final sample size of 8. Figure 2 describes the recruitment process. The inclusion criteria were as follows: > 18 years of age; on dialysis for > 3 months and urinary outputs of < 200 ml/day by self-report. Exclusion criteria included history of diabetes mellitus, pre-dialysis serum phosphate > 2.5 mmol/L and serum potassium > 6.3 mmol/L on most recent routine monthly blood test; parathyroidectomy; history of calciphylaxis; and acute concurrent illness.

The study took place at a clinical research facility (CRF), in a university hospital close to the dialysis unit on the day between two dialysis sessions e.g. a patient who dialysed on Monday and Wednesday participated in the trial on a Tuesday. Each participant attended the (CRF) on two separate days, one week apart.

On the feeding days, participants were directly observed eating the standard low phosphorous diet or the modified low phosphorus diet, depending on the allocation.

The research dietitian (FB) provided the catering department with recipes and an individualised meal plan for each participant and supervised the weighing and plating of the meals. Assignment to each order e.g. standard followed by modified was not randomised. However catering staff were blinded to the identity of the participants and they decided on the order of the allocation e.g. standard diet on day 1 based on hospital food supplies. Participants and researchers were blinded to the allocation. However, once the meals were seen, it was obvious which contained the foods not usually permitted. Participants were asked to attend the CRF between 8 and 9 am having completed an overnight fast and were requested not to take their phosphate binders on the day of the study. Adherence was assessed by asking the participant to confirm whether or not they took their binders that morning. On the feeding day, participants were directly observed taking their meals and medications.

Upon arrival at the unit participants had an initial blood draw and breakfast was served between 8.45am and 9am. Two participants had a planned late arrival on both weeks and so were absent at the initial fasting blood draw. They were provided with a packed breakfast to have on the morning of the study prior to attending the research unit. All participants had a pre-prandial blood draw immediately before a lunch time main meal, which was served between 12 noon and 12.30pm. At each timepoint we took 2 samples, a serum (volume =4.9mL) for the potassium and phosphate analysis and an ethylenediaminetetraacetic acid (EDTA) sample (volume = 2.7mL) for the iPTH analysis. Bloods were collected at hourly intervals following completion of the lunch time main meal for 4 hours (1, 2, 3, 4 hours), and a light evening meal was provided prior to departure from the unit. This procedure was repeated the following week, where participants received the alternate diet plan. All serum samples were spun on a Nuve Bench top Centrifuge Model NF200 (spinning conditions unknown) and frozen at -80°C and subsequently analysed by the Biochemistry Department at the University Hospital. A pre dialysis sample was taken the day after the study. Phosphate was analysed using a photometric UV test for the quantitative determination of inorganic phosphorous in human serum using the ammonium molybdate method on a Beckman Coulter analyser ^31, 32^.

Potassium was measured using the ion selective electrode module of Beckman Coulter AU analysers for the quantitative (indirect) determination of Potassium (K+) concentrations in human serum ^33^.

iPTH was measured using the ARCHITECT Intact PTH assay is an *in vitro* chemiluminescent microparticle immunoassay (CMIA) for the quantitative determination of intact parathyroid hormone (PTH) in human serum on the ARCHITECT iSystem ^34^.

As part of standard care monthly pre-dialysis blood samples were routinely taken and analysed in the same laboratory. Pre-dialysis phosphates from three months before and three months after the study were collected following the study to further contextualise the variability in baseline phosphate levels seen in the study.

### Interventions

Table 1 describes the two diets. The substantial difference between prescribed the diets was at the lunch time main meal. The standard low phosphorus diet ^35^ main meal contained a higher proportion of foods with a higher phosphorus to protein ratio (salmon and dairy), and foods with higher phosphorus bioavailability (Madeira cake). The modified diet, representative of the proposed modified phosphorus diet used food of lower phosphorus to protein ratio such as beef and less dairy. When consuming the lunch time main meal, participants were advised to focus on the protein and phosphorus sources namely soup, beef, milk and nuts for the modified diet and salmon, milk and cake for the standard diet. Whilst in usual clinical practice, patients are advised to spread their dietary allowances over the day, on special occasions such as a wedding, for example, they are permitted to keep their allowances for a single meal. On this basis, 80% of the protein allowance was prescribed at the main meal. Food that was not consumed was weighed to ensure accurate assessment of food intake. Nutrient analysis was conducted using Nutritics software Research Edition v5.028 ^36^. using the McCance and Widdowson's composition of foods integrated dataset ^37^. The potassium data was adjusted to account for leaching of potassium by double boiling the potatoes ^38, 39^. Food samples were sent to the Public Analyst Laboratory, (Cork, Ireland) for analysis of total phosphorus. A spectrophotometric method was used in which the food samples were dry ashed in the presence of Zinc Oxide and total Phosphate content was measured colourimetrically as Molybdenum Blue at 880 nm ^40^.

**Table 1.** Description of the standard low phosphorous diet and the modified low phosphorous diet.

	Standard Diet (S) (based on current management)	Modified Diet (M) (based on proposed new diet sheet)
**Light Breakfast**	Tea & 15ml milk 100 ml orange juice 2 slices white bread with butter & marmalade	Tea + 35ml milk 100 ml orange juice 1 slice white bread & 1 slice wholemeal toasted with butter & marmalade
**Lunch Time Main Meal**	250 ml milk *Steamed Salmon & white sauce 200g potatoes (double boiled) 100g carrots (boiled) 75g broccoli (boiled) 60g Madeira cake	130 ml milk Chickpea soup (100g pulses) *Roast Beef & gravy 200g potatoes (double boiled) 120g carrots (boiled) Meringue (20g) with strawberries (90g) & cream (35g) 25g peanuts
**Evening Meal**	Tea & 15ml milk 2 slices white bread with butter & jam Apple	Tea & 35 ml milk 1 slice white bread 1 slice wholemeal bread with butter & jam

*The salmon and beef portions were individualised to achieve a prescribed intake of 1.1g protein/kg ideal body weight per day for each participant. Approximately 30% dietary phosphorus in the modified diet came from foods with significant phytate content such as pulses, nuts and whole grains.

 Participants were advised to revert to their regular dietary routine between the interventions. For the second feeding day (+ one week), participants were again asked to attend the CRF between 8 and 9 am having completed an overnight fast and were requested not to take their phosphate binders on the day of the study

### Outcomes

Outcome measures were serum phosphate, potassium and iPTH, obtained: early morning (fasting), pre-prandial; at 1,2,3 and 4 hours post lunch time main meal consumption; and pre-dialysis the following day.

### Statistical analysis

Given that this study served as a pilot, we did not calculate the sample size that would be required to reliably estimate the effect of the modified diet plan on patient outcomes. This follows from established best practices in pilot and feasibility studies ^41^ for which there is usually too much uncertainty in the various factors that are needed to make a sample size calculation robust. Our sample size justification is instead based on trying to recruit the largest possible sample given existing financial and pragmatic constraints. The effect of diet on serum phosphate and serum potassium levels was examined using a mixed effects model with diet and time as fixed effects and the patient as a random effect on the intercept as outlined by Bates *et al*., through use of the linear mixed effects regression (lmer) function contained within the lme4 package (version 1.1-21) ^42^. An interaction term between time and diet was included in both models. The difference between mean serum levels following the standard and modified diets at each time point was tested using the general linear hypotheses function (ghlt) from the multcomp package (version 1.4-10) ^43^. Differences in phosphorus content of foods between estimation methods were assessed using paired t-tests. All statistical analyses were performed using R version 3.6 ^44^.

## Results

The baseline characteristics of the 8 enrolled participants are as follows: mean (SD) age was 66.6 (7.8) years ranging from 55.9 to 77.1 years. The mean (SD) body mass index (BMI) was 25.2 (4.0) kg/m ^2^ ranging from 17.9 to 31.3. The median (min, max) pre-prandial iPTH (ng/l) when consuming the standard diet was 294 (114, 663) and when consuming the modified diet was 377 (182, 775). Their dialysis vintage was 52 months ± 46.4, ranging from 19 to 164 months. There were seven males and one female. Four had a fistula and four had a line for dialysis access.

Overall, five participants commenced on the modified diet and three commenced on the standard diet. One male participant, whilst assigned to the modified diet, ate two wheat biscuits with 100mls of milk prior to arrival, and his early morning blood test was therefore omitted from the analysis. Participants took an average of 41 minutes to consume the standard diet lunch time main meal and 37 minutes to consume the modified diet lunch time main meal. Additionally, a study deviation from the protocol was noted for participant 8, who ate in a fast food restaurant after leaving the research unit; therefore his pre-dialysis sample for the following day was excluded from the analysis.

**Figure 3.**
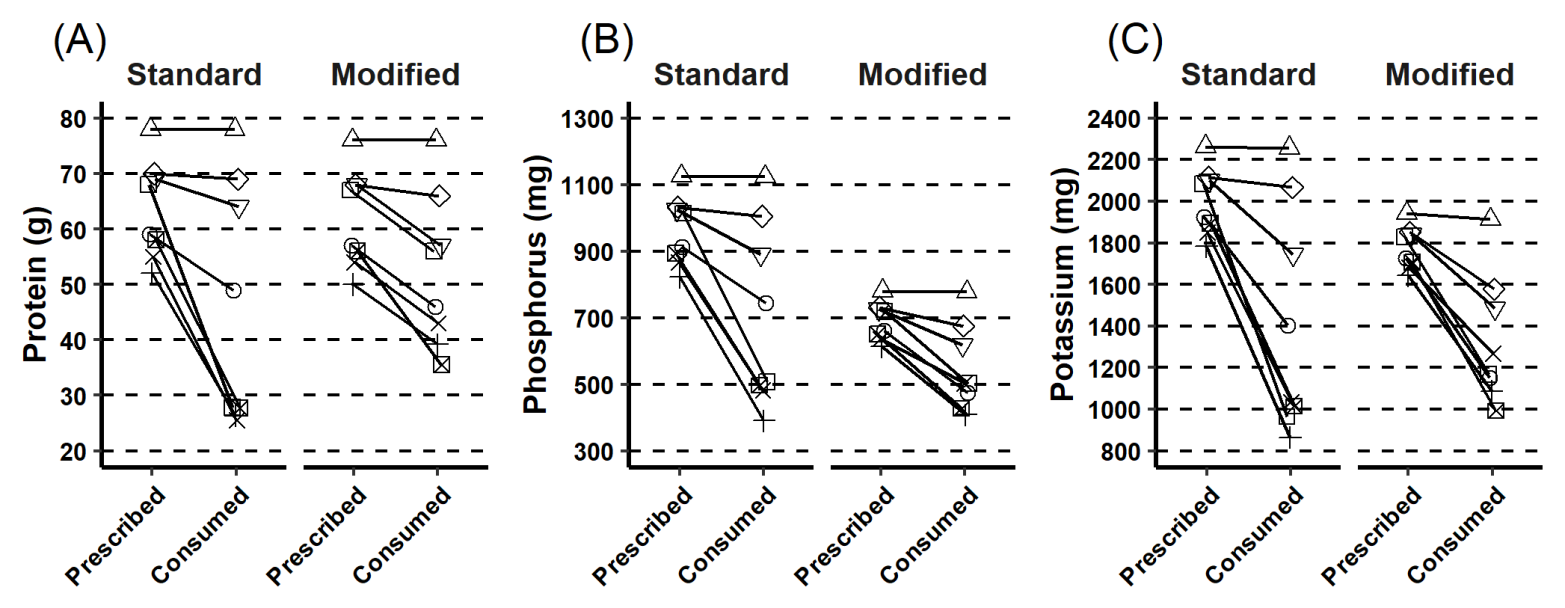
Protein, phosphorus and potassium intake. This figure describes the prescribed and consumed intake for each individual for the standard and modified lunch time main meal for ( **A**) protein, ( **B**) phosphorus and ( **C**) overall. Participants consumed less than prescribed.

Compared to the standard low phosphorous diet, participants consumed a mean of 6.4g more protein per day (p = 0.185, 95% confidence interval [CI]; -3.9, 16.8); 156 mg less phosphorus (p = 0.029, 95% CI; -292.4, -20.9) and 88.6mg (2.3mmoles) less potassium (p = 0.41, 95% CI; -327.6, 150.3) on the modified low phosphorus diet ( Figure 3). On the modified low phosphorous diet participants consumed 7.0 g more fibre (p < 0.001, (95% CI; 5.8, 8.2).

**Figure 4.**
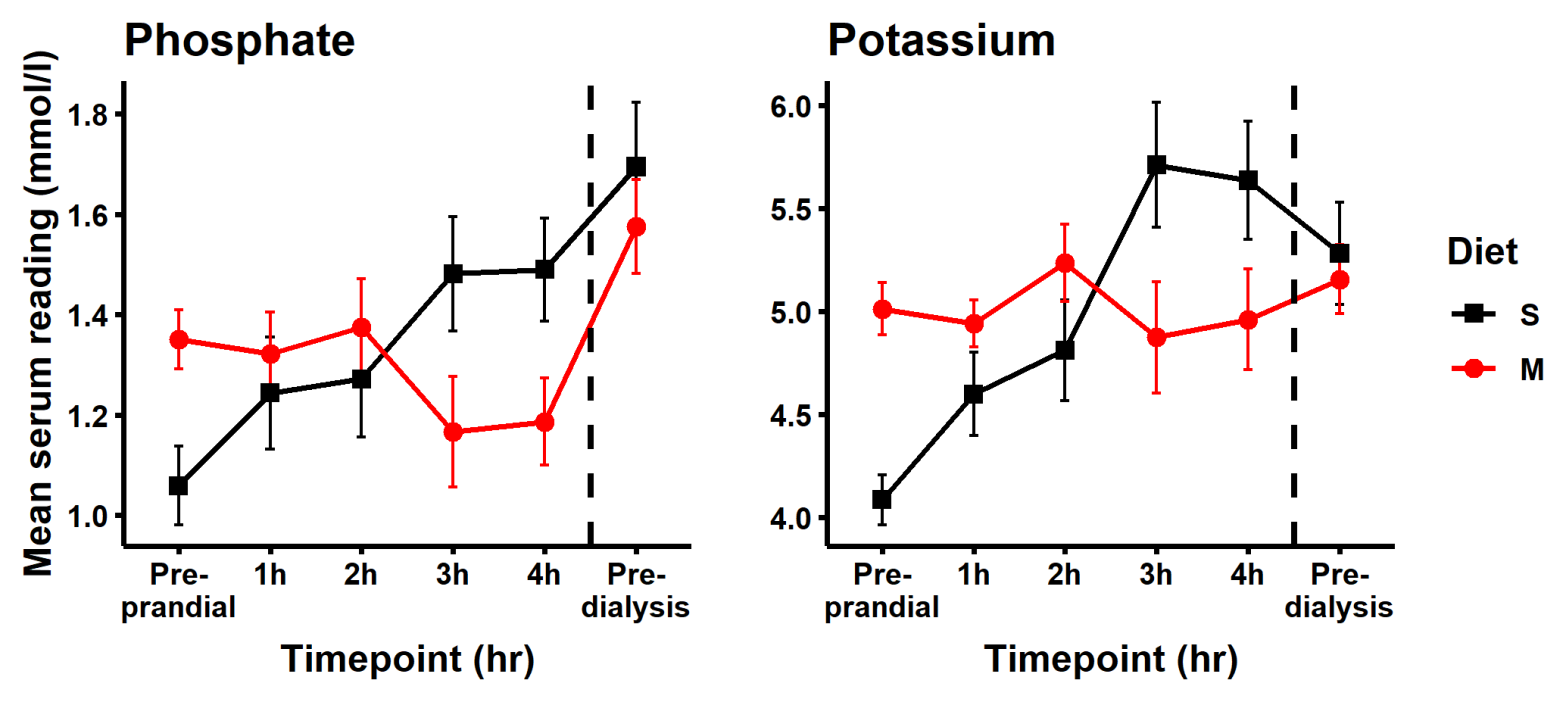
Serum phosphate and potassium levels. Post prandial serum readings for participants on standard low phosphorus diet S (black line with square) increased steadily over time and remained elevated the following day. This contrasted with an overall decrease in serum phosphate following consumption of the modified diet main meal (M; red line with circle). Points represent the mean ± standard deviation (SD).

**Figure 5.**
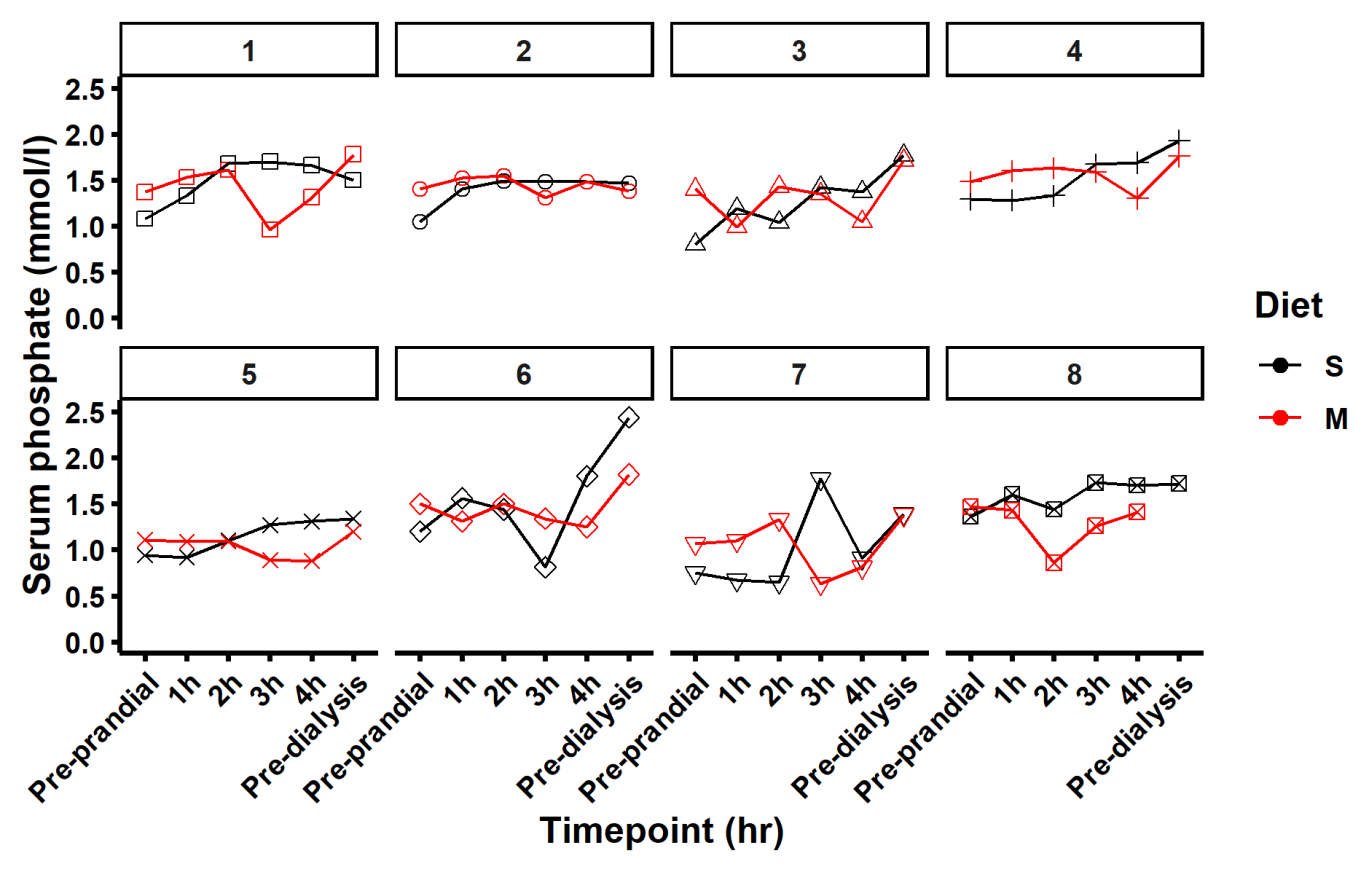
Serum phosphate levels. Figure 5 maps serum phosphate for each participant on the diet standard (S) in black and modified (M) in red at 6 time points pre-prandial, 1, 2, 3 and 4 hours after the meal and pre dialysis the following day.

The pre-prandial time point data indicated that participants when assigned to the modified diet had a mean starting phosphate serum reading of 0.291 mmol/L higher than when assigned to the standard diet ( Table 2 and Figure 4, (95% CI: 0.026, 0.556). Similar between group differences were seen in the fasting samples taken on arrival. The mean change in serum phosphate from pre-prandial levels was smaller at all four time points on the modified diet relative to the standard diet. Using a mixed effect model to describe the change in serum phosphate when switching from the standard to the modified diet, we observed significant mean reductions in serum phosphate at 3 hours, -0.32 mmol/L (95% CI: -0.58, -0.05), and at 4 hours, -0.30 mmol/L (95% CI -0.57, -0.04), Table 2. Figure 5 shows the phosphate data of each subject individually and Figure 4 shows the group mean for pre-prandial and each of the four hourly measurements of serum phosphate post-meal and describes an elevation in serum phosphate following consumption of the standard diet and an overall decrease in serum phosphate following consumption of modified diet.

**Table 2.** Mixed effects model output of serum phosphate and potassium.

Time point	Mean mmol/L	Std. Error	p-value	95% Confidence Interval	
				Lower	Upper
Pre-prandial serum phosphate	0.291	0.101	**0.023**	0.026	0.556
1 h serum phosphate	0.078	0.101	0.970	-0.187	0.342
2 h serum phosphate	0.104	0.101	0.885	-0.161	0.369
3 h serum phosphate	-0.315	0.101	**0.010**	-0.580	-0.050
4 h serum phosphate	-0.304	0.101	**0.015**	-0.569	-0.039
Pre-dialysis serum phosphate	-0.104	0.104	0.901	-0.379	0.171
Pre-prandial serum potassium	0.975	0.226	**0.001**	0.381	1.569
1 h serum potassium	0.363	0.226	0.494	-0.230	0.957
2 h serum potassium	0.425	0.217	0.268	-0.147	0.997
3 h serum potassium	-0.838	0.217	**0.001**	-1.410	-0.265
4 h serum potassium	-0.675	0.217	**0.011**	-1.247	-0.103
Pre-dialysis serum potassium	-0.098	0.243	0.999	-0.737	0.540

The mixed effect model describes the change in serum phosphate and potassium when switching from the standard to the modified diet. Fixed effects were diet and time point which included an interaction term, with patient included as a random effect.

**Figure 6.**
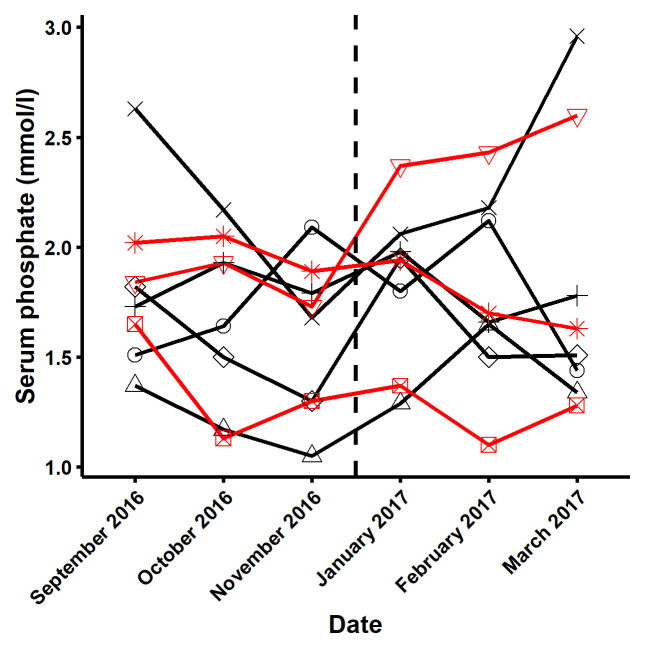
Within person variability in pre-dialysis serum phosphate. This figure describes the variability, in the three months before and after the study in the three participants who started with the standard diet (black line) and the five participants starting with the modified diet (red line).

To further evaluate the differences seen in pre-prandial baseline phosphate levels between the modified vs. the standard diet, we examined the pre-dialysis phosphate levels measured in routine testing in the three months prior to and following the study in a *post hoc* analysis. These measurements showed substantial within subject variability ( Figure 6) and were compatible with the differences in baseline values seen within participants in the study. The mean (SD) pre and post study, routinely measured serum phosphate levels were similar in those assigned initially to standard diet (1.75 ± 0.42 mmol/L, range 1.05 – 2.96) versus modified diet (1.78 ± 0.44 mmol/L, range 1.10 – 2.60) respectively, in keeping with our interpretation that the differences in the point estimates of baseline serum phosphate were consistent with the inherent variability in serum phosphate. The small sample size and high variability in iPTH analysis, made it impossible to interpret the data.

**Figure 7.**
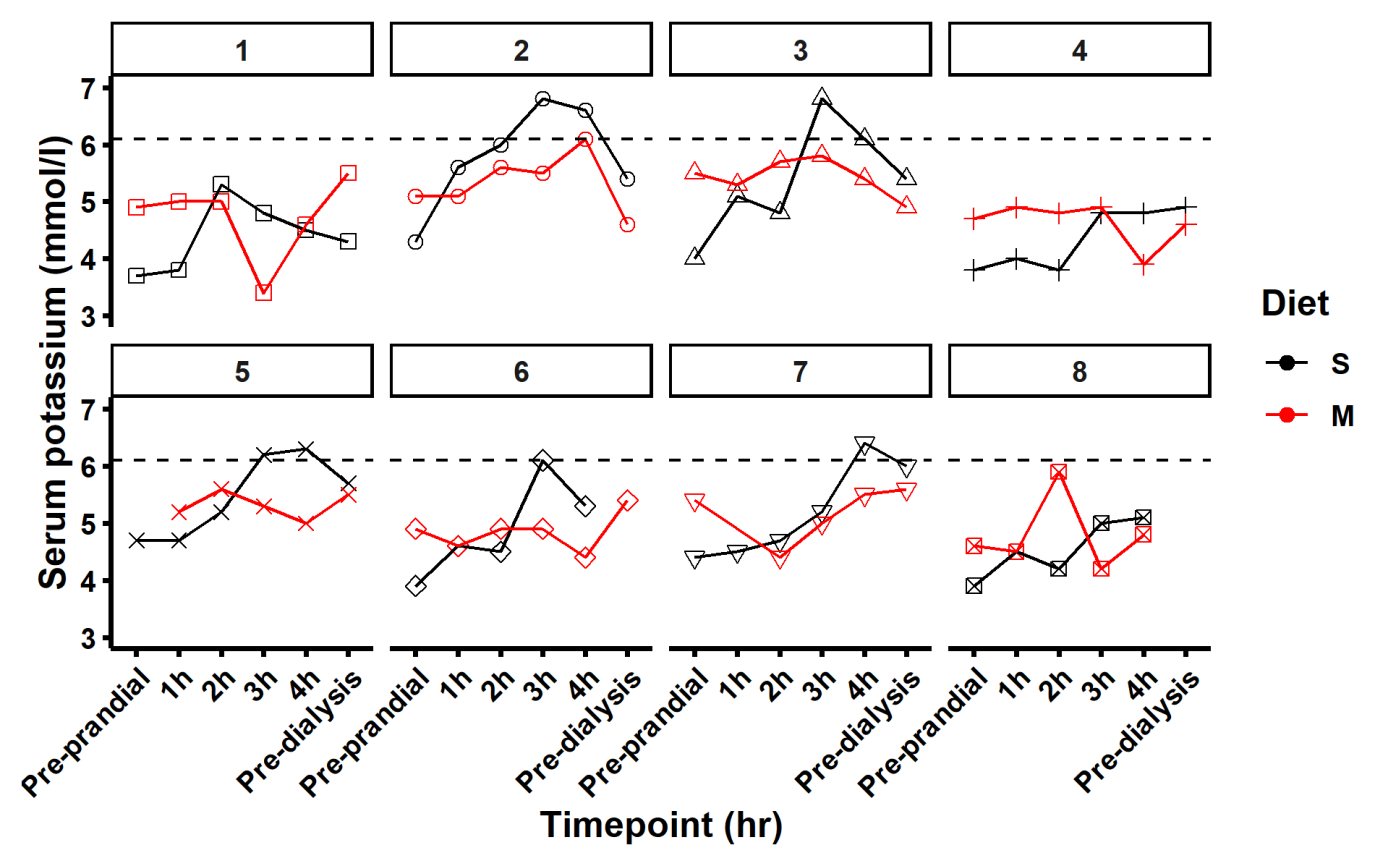
Serum potassium levels. Figure 7 maps serum potassium on each diet standard (S) in black and modified in red (M) at 6 time points pre-prandial, 1, 2, 3 and 4 hours after the meal and pre dialysis the following day. Five participants had a potassium reading of > 6.0 mmol/L (dashed line) during the study timeframe. Eight of the nine elevated readings were recorded in the standard diet cycle of the study.

When switching from the standard to the modified diet, serum potassium decreased by -0.84 mmol/L (95% CI: -1.41, -0.27) and -0.68 mmol/L (95% CI -1.25, -0.103), at the three- and four-hour post-meal time points, respectively ( Table 2 and Figure 4). Nine samples were recorded with serum potassium > 6.0 mmol/L and were classified as hyperkalaemic. Of these, eight were from participants while on the standard diet ( Figure 7). This hyperkalaemia was transient with no participant remaining hyperkalaemic the following day pre dialysis. While consumed dietary potassium intakes were similar on both the modified and standard diets, serum potassium was stable or reduced following consumption of the modified meal and steadily increased for 3 hours after consumption of the standard meal ( Figure 4). Individual level biochemical and dietary intake data is available online at the Open Science Framework ^45^.

**Figure 8.**
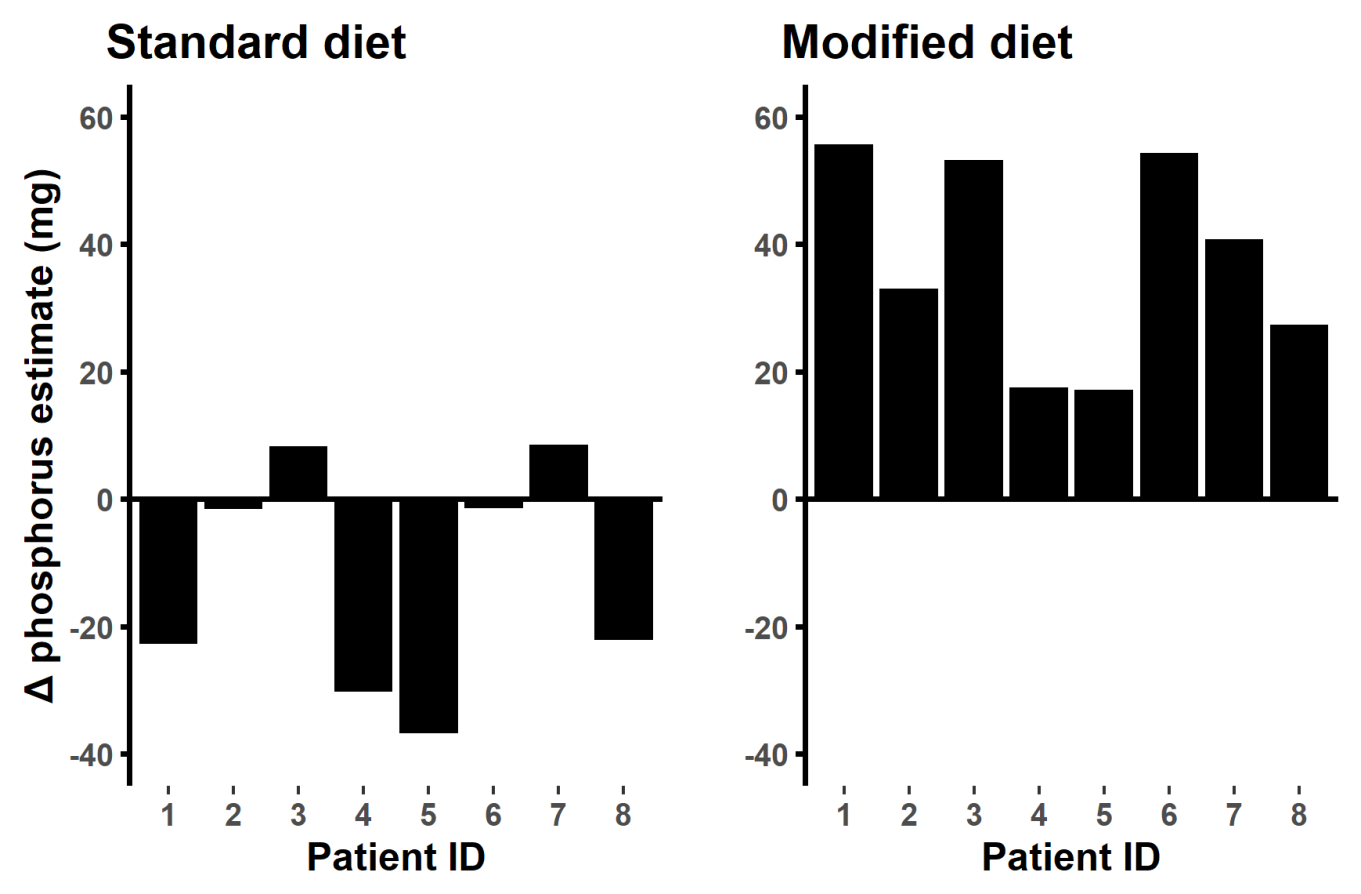
Laboratory analysis versus food composition table estimates. Difference in food composition table reading minus public lab reading. On the standard diet the food tables estimate of daily intake were similar to the laboratory analysis (p= 0.148) while on the modified diet the food table overestimated the phosphorus content by 37.4mg/day (p= 0.008).

The food composition tables provided a similar estimate of phosphorus content to chemical analysis for the standard diet food samples whereas the food composition tables overestimated phosphorus by 37 mg/ day for the modified diet food samples (p < 0.001, 95% CI 24,51) ( Figure 8).

## Discussion

In this study consumed dietary intake of phosphorus was lower on the modified low phosphorous diet, while dietary potassium and protein intakes were similar in both diets. Both serum phosphate and serum potassium were reduced at 3 and 4 hours on the modified.

Focusing firstly on serum phosphate, the observed lower serum phosphate is consistent with the decreased dietary phosphorus load consumed in the modified diet, because it contained foods with lower phosphorus to protein ratios. In addition the food matrix, specifically the lower bioavailability of phosphorus from the phytate sources of pulses and nuts, will likely have contributed to reduced absorption of phosphorus and the observed lower serum phosphate ^11, 24^. Other factors that may have influenced serum phosphate were circadian pattern and mineral and bone disease status. Physiologically, serum phosphate has a circadian pattern ^12– 16^ although the responsible mechanisms are poorly understood ^46^. However in the study circadian patterns were controlled, as food was served at the same time on each trial day and therefore we do not believe this unduly influenced the comparison of the standard and modified low phosphorus diet. Similarly, whilst mineral and bone disease status can also affect serum phosphate levels, baseline levels indicate that tertiary hyperparathroidism should not have unduly influenced the results and each participant consumed both diets with an adequate washout period, thus controlling for this variable.

We demonstrated that whole food as distinct from phosphate supplements can affect phosphate in the postprandial phase. There are a limited number of small studies in this field. In a recent study by Lundin *et al*. in 12 HD patients, a meal with high phosphorus content did not significantly affect plasma phosphate compared to a meal with low phosphorus content ^47^. Isakova *et al*. examined the difference between 2 whole-food meals of 250 mg and 500 mg of phosphorus content in 13 patients with CKD stage 3 to 4, and observed that serum phosphate decreased after both meals. In a feeding study on 30 HD patients, a meal containing 1g calcium and 2g phosphorus as a phosphorus supplement, did not increase postprandial serum phosphate ^22^. Preliminary experiments by Brown *et al.* with whole food failed to result in significant increases in serum phosphate, however, when a drink with a phosphorus supplement (1280mg) was used , serum phosphate was significantly increased at 2 and 4 hours (n=8) ^23^. Our results support the finding by Ix *et al*. ^14^ in 11 CKD patients that whole food intake can affect serum phosphate. In a longer study that included post prandial observations on the final day, Moe *et al.* demonstrated a vegetarian diet in 9 patients with advanced CKD led to lower serum phosphorus levels than a meat based diet ^48^.

Two questions emerge as we consider the results of our study and similar studies, firstly the effect of timing of the intervention on post prandial blood levels and secondly the relative importance of post prandial versus fasting levels. The timing of the intervention may have greater than previous appreciated effect on the serum phosphate levels. The first three studies which were negative were conducted in the morning ^22, 47, 49^. The study by Brown *et al*. was conducted in the morning and failed to show results with solid foods but did lead to an increase in serum phosphate with a large dose of a phosphate supplement ^23^. Both Ix *et al* and Moe *et al* monitored bloods for 24 hours and demonstrated greater change and greater response of serum phosphate to diet in the afternoon ^14^ or evening ^48^. We demonstrated that food intake can alter serum phosphate in the afternoon. Given that serum phosphate exhibits a marked circadian rhythm, with a peak around 3:00 am and a nadir around 11:00 am ^50^, we suggest that this nadir may mask the effect of dietary phosphorus load in the morning.

This leads to a second related question namely the relative importance of post prandial versus fasting levels of serum phosphate. Our study focused on the effect of diet on post-prandial serum phosphate. In healthy populations dietary phosphorus load causes serum phosphate to rise ^15, 17, 18, 51– 53^ but does not affect subsequent fasting levels ^15, 19, 53^ with the exception of cheese in one study ^53^. Similarly in the CKD population fasting serum phosphate levels have been shown to be a poor indicator of phosphorus load ^14^ even though fasting levels may be a more reliable predictor of mortality ^54, 55^.

Potential mechanisms of harm have been identified for both non-fasting and fasting serum phosphates. A phosphorus load that induces postprandial hyperphosphataemia impairs endothelial function ^17, 18^ while a phosphorus load sufficient to raise subsequent fasting phosphate, significantly increases blood pressure and pulse ^56^. While experts suggest research efforts should be focused on altering fasting levels which may be most harmful ^57^, there is still merit in focusing on postprandial measures where effects of diet are more evident ^14^ and where we have an identified potential mechanisms of harm ^17, 18^. Our study did not collect subsequent fasting levels but did collect a pre dialysis sample the following day that was not significantly different between the groups. Whilst we need a better understanding of the relative clinical and prognostic importance of fasting versus postprandial versus pre dialysis serum phosphate, this study provides us with some insight into the effect of diet on the post prandial period and demonstrates the potential weakness of pre dialysis measures of serum phosphate which are often taken early morning or at lunch time and are routinely used to guide clinical treatment. Our finding in the context of similar studies also suggest that future studies should be carried out in the afternoon.

Despite similar dietary potassium intakes on the standard and modified diet, serum potassium was reduced at 3 and 4 hours post-meal consumption in participants who consumed our modified low phosphorus diet compared with the standard low phosphorus diet. This suggests that the food matrix of the modified diet foods may also reduce the absorption of potassium. There is limited evidence from animal studies the phytate content may reduce potassium absorption ^58^. Whilst potassium absorption is poorly understood, it has been suggested that the higher potassium load of plant-rich diets may be offset by biological factors affecting potassium metabolism and that dietary fiber may reduce potassium absorption ^59^. We also observed a transient hyperkalaemia in five participants on the standard diet during the postprandial period that had resolved by the time a pre dialysis sample was taken the following day ( Figure 7). This is of significance as we rarely check postprandial potassium levels and might underestimate the magnitude of the postprandial spike. This result also indicates that caution may be required in permitting patients to save their dietary allowances for one meal, as with special occasions, and it may be more prudent to spread allowances over the day, from both a protein utilisation and potassium load safety viewpoint ^60^.

In this study, the food composition tables overestimated the phosphorus content of the low phosphorus modified diet in comparison to direct analysis and similarly estimated the phosphorus content of the standard diet ( Figure 8). This conflicts with concerns among renal health care professionals that food composition tables underestimate the phosphorus content of foods. This may be partially explained by the fact that most of the food consumed in this study was fresh and unprocessed and the concern from renal healthcare professionals is largely focused on processed foods. In keeping with a study by Tsai *et al.,* ^61^ our results, provide reassurance regarding the reasonable reliability and accuracy of food composition tables, especially with regard to unprocessed foods.

There were some limitations to this study including a small sample size, gender imbalance and short duration. There was unexpected pronounced difference between the baseline pre-prandial phosphate levels of participants when assigned to the modified and the standard diet, a potential issue for any highly variable parameter with a small sample size. The differences in phosphate levels may have occurred simply due to sampling variability, given the small sample size and substantial variability in the within subject serum phosphate level, which we demonstrate in a *post hoc* review of the routine dialysis bloods of the study participants before and after the study ( Figure 6) and as reported by other studies ^62, 63^. However this variability should not substantially impact on the additional effect of diet relative to the measured baseline, especially in the setting of a short term follow-up of serum phosphate levels in the immediate postprandial hours, where the environment, type and timing of the food, biochemical assays and medications were all strictly controlled. A strength of this study is that it was conducted in a highly controlled setting, with a defined diet and timed regular blood draws. A second strength is that we tested foods and normal meals from our current standard low phosphorus diet and compared it to foods from a proposed modified low phosphorus diet.

This feeding study supported the postprandial safety and tolerability of the modified versus the standard diet, and allowed for further evaluation of the modified diet a randomised controlled trial of 74 patients over one month which concluded that the diet was well tolerated and was associated with similar phosphate and potassium control but with a wider food choice and greater fiber intake than the standard diet ^64^. This feeding study should be repeated, with larger numbers in the afternoon or evening. With numbers of people worldwide with kidney disease estimated at 850 million ^65^, and limited evidence to guide our practice ^10, 66^ we need to continue to undertake clinical trials in the field of nutrition to help our understanding of the effects of diet in CKD ^59, 67^.

## Conclusion

Our understanding of gastrointestinal absorption of phosphorus is surprisingly limited and requires further investigation. The modified diet included more wholegrains, pulses and nuts with lower phosphorus bioavailability, food choices with a lower phosphorus to protein ratio and the avoidance of all phosphate additives. On the modified diet, the serum phosphate and potassium levels were significantly lower at 3 and 4 hours postprandial suggesting reduced absorption of these nutrients.

## Data availability

### Underlying data

Open Science Framework: The Effect of a Dietary Phosphorus Load and Food Matrix Change in a Controlled One Day Feeding Trial. https://doi.org/10.17605/OSF.IO/M3AUQ ^45^. 

This project contains the following underlying data:

- Data_dictionary.csv
- Diets.csv
- Main_data.csv

Data are available under the terms of the Creative Commons Attribution 4.0 International license (CC-BY 4.0).
