# Supplementary material for: The effect of dietary phosphorus load and food matrix on postprandial serum phosphate in hemodialysis patients: a pilot study
Source: HRB Open Res. 2021 Nov 10;4:119. [Version 1] doi: 10.12688/hrbopenres.13382.1 (PMC8822142; doi:10.12688/hrbopenres.13382.1)
Supplement: Peer review history

# Peer review: Reviewer response for version 1

**DOI:** 10.21956/hrbopenres.14576.r30860

**Published:** 2022-02-07

**Authors:** Donal Sexton

**Affiliations:** 1 St James's Hospital, School of Medicine, Trinity College Dublin, Dublin, Ireland

Thank you for the opportunity of reviewing this study. Overall I find the methodology of this study very comprehensive, and possesses considerable internal validity with the caveat of the small sample size. The study protocol is very well defined, and limitations in allocation concealment and sequence randomization acknowledged. Contamination from co-interventions such as phosphate binders is also considered in the protocol, although perhaps vitamin D intake (and its effect on serum phosphate could also have been included in a contamination protocol). Does fasting have an effect on serum phosphate other than reducing phosphate intake? For example insulin induced re-distribution? Presumably this would have been accounted for by the standardized length of fasting for participants. 

 The statistical analysis are appropriate and the results and figures clearly and concisely reported. The addition of an analysis of pre-dialysis phosphate levels measured in routine care 3 months pre and post the study is adds to the study findings. 

 The comparison of public lab based analysis of phosphate content and estimated content is very interesting, particularly for the modified diet. 

 The conclusions are appropriate in my opinion and discuss alternative explanations of the study findings as well as existing literature on the topic. As acknowledged by the authors a longer study with multiple cross over  periods between standard and modified diets would be welcomed however it may not be practically achievable due to the resources necessary.

Is the work clearly and accurately presented and does it cite the current literature?

Yes

If applicable, is the statistical analysis and its interpretation appropriate?

Yes

Are all the source data underlying the results available to ensure full reproducibility?

Yes

Is the study design appropriate and is the work technically sound?

Yes

Are the conclusions drawn adequately supported by the results?

Yes

Are sufficient details of methods and analysis provided to allow replication by others?

Yes

Reviewer Expertise:

Nephrology, Randomised Trials, Data Science.

I confirm that I have read this submission and believe that I have an appropriate level of expertise to confirm that it is of an acceptable scientific standard.

# Peer review: Reviewer response for version 1

**DOI:** 10.21956/hrbopenres.14576.r30861

**Published:** 2021-12-06

**Authors:** Jordi Bover

**Affiliations:** 1 Hospital Germans Trias i Pujol (HGTiP), REMAR group, RICORS, Institut de Recerca Germans Trias i Pujol (IGTP), Badalona (Barcelona), Catalonia, Spain

This is an excellent new contribution of a well known Irish group in the field of nutrition in patients with chronic kidney disease (CKD). Diet in CKD patients is one of the mainstays of their overall treatment and, in this study, authors do not only provide new information on the acute postprandrial effect of a previously described modified versus a standard low phosphorus (P) diet on P levels but also on potassium (K). This piece of information is very valuable because some concerns were risen on the effects of such diet modification from previous reports. 

 Authors conducted a nice controlled crossover feeding study in 8 hemodialysis patients (one female)  and samples were collected at one-hour intervals for the four hours after the lunch time main meal for analysis. They observed that individuals on the MODIFIED diet had serum P AND K readings lower at 3 and 4 hours than when on the standard diet, and nicely discuss their results by comparing them with a limited number of other small studies previosuly reported. Importantly, authors do not only underline the important differences between fasting and postprandrial measurements of these ions, but also stress the frequently overseen importance of circadian rhythms affecting divergent results in different studies. 

 I only miss a discussion on the potential effect of unbalanced sex (only one female) in their observations, a clear evolution of calcium and PTH values (trade-off hypothesis revisited in humans?) and the potential contribution (although unlikely significant) of FGF23 in an acute setting.

Is the work clearly and accurately presented and does it cite the current literature?

Yes

If applicable, is the statistical analysis and its interpretation appropriate?

Yes

Are all the source data underlying the results available to ensure full reproducibility?

Yes

Is the study design appropriate and is the work technically sound?

Yes

Are the conclusions drawn adequately supported by the results?

Yes

Are sufficient details of methods and analysis provided to allow replication by others?

Yes

Reviewer Expertise:

Chronic Kidney Disease- MIneral and Bone disorders (CKD-MBD)

I confirm that I have read this submission and believe that I have an appropriate level of expertise to confirm that it is of an acceptable scientific standard.
